# Real-Time Infoveillance of Moroccan Social Media Users’ Sentiments towards the COVID-19 Pandemic and Its Management

**DOI:** 10.3390/ijerph182212172

**Published:** 2021-11-19

**Authors:** Abdelghani Ghanem, Chaimae Asaad, Hakim Hafidi, Youness Moukafih, Bassma Guermah, Nada Sbihi, Mehdi Zakroum, Mounir Ghogho, Meriem Dairi, Mariam Cherqaoui, Karim Baina

**Affiliations:** 1 TICLab, College of Engineering & Architecture, International University of Rabat, Rabat 11103, Morocco; chaimae.asaad@uir.ac.ma (C.A.); hakim.hafidi@uir.ac.ma (H.H.); youness.moukafih@uir.ac.ma (Y.M.); bassma.guermah@uir.ac.ma (B.G.); nada.sbihi@uir.ac.ma (N.S.); mehdi.zakroum@uir.ac.ma (M.Z.); mounir.ghogho@uir.ac.ma (M.G.); 2 École Nationale Supérieure d’Informatique et d’Analyse des Systèmes, Mohammed V University, Rabat 10000, Morocco; karim.baina@ensias.um5.ac.ma; 3 College of Management, International University of Rabat, Rabat 11103, Morocco; meriem.dairi@uir.ac.ma; 4 University Ibn Tofail, Kenitra 14000, Morocco; mariam.cherqaoui@uit.ac.ma

**Keywords:** COVID-19, emotion analysis, machine learning, polar sentiment analysis, topic modeling, universal language model for Moroccan dialect

## Abstract

The impact of COVID-19 on socio-economic fronts, public health related aspects and human interactions is undeniable. Amidst the social distancing protocols and the *stay-at-home* regulations imposed in several countries, citizens took to social media to cope with the emotional turmoil of the pandemic and respond to government issued regulations. In order to uncover the collective emotional response of Moroccan citizens to this pandemic and its effects, we use topic modeling to identify the most dominant COVID-19 related topics of interest amongst Moroccan social media users and sentiment/emotion analysis to gain insights into their reactions to various impactful events. The collected data consists of COVID-19 related comments posted on Twitter, Facebook and Youtube and on the websites of two popular online news outlets in Morocco (Hespress and Hibapress) throughout the year 2020. The comments are expressed in Moroccan Dialect (MD) or Modern Standard Arabic (MSA). To perform topic modeling and sentiment classification, we built a first Universal Language Model for the Moroccan Dialect (MD-ULM) using available corpora, which we have fine-tuned using our COVID-19 dataset. We show that our method significantly outperforms classical machine learning classification methods in Topic Modeling, Emotion Recognition and Polar Sentiment Analysis. To provide real-time infoveillance of these sentiments, we developed an online platform to automate the execution of the different processes, and in particular regular data collection. This platform is meant to be a decision-making assistance tool for COVID-19 mitigation and management in Morocco.

## 1. Introduction

In late December 2019, Wuhan, the capital city of Hubei province in central China reported the first pneumonia cases of unknown origin. The causative pathogen has been identified as a novel enveloped RNA betacoronavirus. Given the phylogenetic similarity to the previously isolated severe acute respiratory syndrome coronavirus (SARS-CoV), the new virus has been named SARS-CoV-2 [1].

Even though the epidemic began and was geographically focused in mainland China at first, the rate of increase in cases became greater in the rest of the world than inside China on 26 February 2020 [2]. The geographical expansion of the epidemic reached the rest of the world hitting Italy, Iran, Spain and the United States the hardest. The World Health Organization declared COVID-19 a pandemic health emergency on 11 March 2020, as most countries went into sanitary emergency states and implemented various levels of lockdown and social distancing protocols.

As of 25 June 2021, 179 million cases of COVID-19 have been recorded worldwide and the resulting death toll reached 3.88 million [3]. In Morocco, reports put the number of confirmed cases at around 527,147 with 9247 resulting fatalities [3].

Since its emergence, COVID-19 has had a dramatic socio-economic impact on most countries as well as on global public health, food systems and employment [4]. During this time, social media websites have become a necessary tool for information sharing, communication, entertainment and as a free-for-all opinion sharing platform. By becoming an increasingly reliable emotional outlet for communities all over the world [5], social media fulfilled a crucial role in influencing people’s perception of the COVID-19 outbreak and the crisis response strategies put in place by governments.

In the midst of the COVID-19 pandemic, the Moroccan population, similarly to the rest of the world, has lived through a turmoil of emotions reflecting the effect not only of the psychological burden of life during the pandemic [6] but also the emotional impact of the necessary mitigation strategies such as quarantine, lockdown, curfew and social distancing.

The main objective of this study is to investigate how Moroccan social media users are responding to sanitary measures and other regulations as well as their collective emotional reaction to different aspects of life during the COVID-19 pandemic. More specifically, we aim to gauge and analyze the emotional response of Moroccan social media users to COVID-19 and its impact on public health, education, economy and social life. Additionally, we uncover COVID-19 related topics of discussion amongst Moroccan social media users.

To this end, we conducted a year-long data collection campaign on social media platforms such as Facebook and Twitter to gather COVID-19 related comments made by Moroccan social media users and written in Moroccan dialect (MD) and Modern Standard Arabic (MSA). Moreover, we collected user comments in response to news articles on popular online news outlets in Morocco such as Hespress www.hespress.com (accessed on 15 October 2021) and www.ar.hibapress.com (accessed on 15 October 2021). A collection of major events and government announcements during this time period was compiled in order to investigate their impact on the emotions of social media users.

An aggregated dataset from various sources of online comments was constructed and put through an annotation process to label the different topics and emotions relayed by the Moroccan social media users in relation to COVID-19. Several Machine Learning algorithms for Topic Modeling and Sentiment Analysis were then tested on this dataset.

The novelty of the work presented in this paper stems from three main points:

1. Unlike existing work, we collected COVID-19 related comments, expressed by a community (here Moroccans) on social media throughout an entire year. This allowed us to derive meaningful insights on emotional responses to COVID-19 related topics and their evolution throughout the year of 2020;
2. Unlike existing work, we collected COVID-19 related events and government policies and investigated social media users’ reactions during the time of their implementation;
3. We proposed the first Universal Language Model for the Moroccan Dialect ‘Moroccan-Darija-ULM’ (MD-ULM), as will be demonstrated in the Results section. This model outperforms classical machine learning classification methods in three different tasks: Topic Modeling, Emotion Recognition and Polar Sentiment Analysis;
4. We developed an online platform which automates regular data collection, facilitates the collaborative annotation process and produces real-time sentiment analysis. This platform is meant to be used to help decision makers design targeted mitigation strategies for this current pandemic and also for future ones.

The remainder of this paper is organized as follows. Related work is presented in Section 2. Our methodology for the collection of comments from various social media sources and their annotation for topic modeling and emotion classification is presented in Section 3.1. The conceptualization of the first Moroccan Dialect Universal Language Model is detailed in Section 3.2. The temporal evolution of COVID-19 induced emotional responses and topics of interest as well as the results of emotion classification, polar sentiment classification and topic modeling are illustrated in Section 4. Our online platform for the automation of our pipeline and decision making assistance is presented in Section 5. Conclusions are summarized in Section 6.

## 2. Related Work

The surge in COVID-19 cases worldwide and the gravity of its impact on public health have heavily contributed to making COVID-19 the dominant topic of the scientific literature this past year. Numerous studies in various domains have been published and highly cited. One particular scope of interest involves the use of Artificial Intelligence (AI) and Natural Language Processing (NLP) to analyze COVID-19 related content such as scientific articles, social media posts and news headlines.

Within this scope, a set of studies focused on using topic modeling for a better understanding of different aspects of the pandemic. In [7], the authors analyzed Twitter users’ reactions using sequential pattern mining (SPM) techniques. In order to study the main topics of interest on Twitter using Latent Dirichlet Allocation (LDA), the authors of [8] focused on the Italian Twitter community, while the authors of [9] analyzed tweets written in English.

In [10], Biterm Topic Model (BTM) was used to discover and describe user-generated interactions that could be related to COVID-19 symptoms and indications of illness recovery as well as difficulties in access to testing. Other researchers used topic modeling to analyse newspaper articles. For example, in [11], the authors developed a topic analysis system of COVID-19 related news articles in Canada. Furthermore, in [12], the authors employed a topic modeling approach to examine news during the early stages of the outbreak in China.

While the aforementioned studies focused on topic modeling, another set of works aimed to make use of sentiment analysis. The authors of [13] used classical machine learning algorithms such as Naive Bayes and Logistic Regression to classify sentiments expressed in tweets. The authors of [14,15] used more sophisticated deep learning based methods such as long short-term memory (LSTM) and convolutional neural networks (CNN). Furthermore, in [14], the authors study how different cultures reacted to the pandemic based on the predominant sentiment expressed in tweets originating from different countries. In [16], the authors used the CrystalFeel algorithm to assess the predominant emotions of the world population. By analyzing more than 20 million tweets, they observed that over the course of the pandemic, the collective public sentiment changed dramatically from fear to anger.

Some studies combined both topic modeling and sentiment analysis to assess people’s sentiments on multiple aspects of the pandemic. In [17], the authors investigated COVID-19 related news across four countries using Top2vec for topic modeling and RoBERTa for sentiment analysis. In [18], the authors used multiple topic modeling techniques and sentiment analysis algorithms to analyze content from Brazil and USA based twitter accounts. In [19,20], the authors combined LDA for topic modeling with several sentiment analysis techniques to understand public discourse during the pandemic.

Although they have not had the same disastrous impact as COVID-19, our modern society has experienced other large-scale health crises. In fact, we live in a risk society where health, political and individual risks are beyond institutional control. These crises seem to be of increasing concern and we must be prepared as a society to deal with them. In this spirit, some works have been conducted for example to analyze the media coverage on the swine flu virus crisis in order to draw lessons to better manage future crises [21,22]. Our work is part of this framework and aims to provide public opinion and decision-makers with a global view of the issues that concern citizens through the use of Artificial Intelligence and social networks analysis.

## 3. Materials and Methods

### 3.1. Data Collection

**Figure 1:**
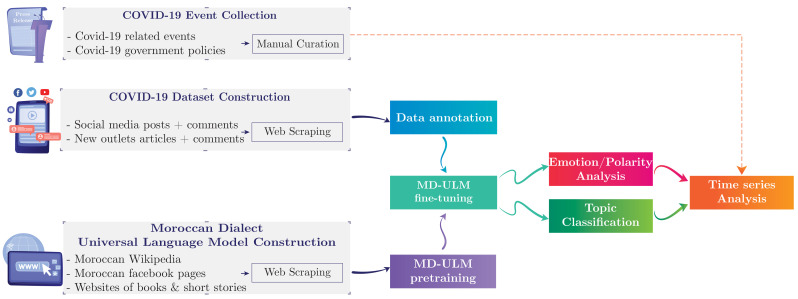
General methodology for COVID-19 Real-Time Infoveillance in Morocco.

In this section, we describe the methodology followed to construct the COVID-19 Moroccan dataset. Figure 1 shows the different components of the proposed methodology, including the Moroccan Dialect Language Model construction process detailed in Section 3.2.

#### 3.1.1. Dataset Construction

In order to gauge the emotional response of Moroccan social media users to COVID-19 related news, we developed a data collection strategy that targeted the most popular social media sites in Morocco. According to Statcounter [23], the most popular social media sites during 2020 in Morocco are Facebook, Youtube, Pinterest and Twitter. Because Pinterest is mainly a bookmarking social media site that enables finding new ideas and planning visual projects (e.g., home improvement, travel agenda), and not necessarily a news and opinion sharing platform, we opted to exclude it from our study. We only include Facebook, Youtube and Twitter as social media sources for comment collection.

To enrich our dataset, we targeted the two most popular digital press websites in Morocco [24]: Hespress and Hibapress, from which we collected user comments on articles related to COVID-19.

The collection was conducted continuously from 1 January 2020 to 13 January 2021 and resulted in a dataset of 747,018 communications.

Tweets, Facebook comments and Youtube comments were collected using their respective APIs: Twitter API, Graph API and Youtube Data API. Web crawlers for Hespress and Hibapress were developed to collect comments from news articles tagged under the COVID-19 news category.

To guide the collection process, the hashtag #Coronavirus_Morocco was used to target tweets made in relation to COVID-19 and Morocco. Prominent public accounts and pages on Twitter, Facebook and Youtube were targeted for comment collection. Examples of these accounts include but are not limited to: Moroccan journalists (e.g., Belhaissi Youssef), Moroccan influencers (e.g., Mustapha Swinga), official government representatives (e.g., Prime Minister of Morocco, Moroccan Ministry of Health) and social media accounts of Hibapress and Hespress.

The comments collected from all these sources were anonymized thus eliminating authorship data, location, images, hyperlinks and non-textual components.

The study universe included around twenty-five million Moroccan citizens, accounting for roughly 69% of Moroccan social media and internet users according to the digital report of Morocco [25]. Each internet user in Morocco had the same odds of being included in this research.

To satiate the requirements of our study, we compiled a list of inclusion and exclusion criteria to be applied on each record of our collected dataset.

To be included, the communication must:

- Make an implicit or explicit reference to the COVID-19 pandemic in Morocco.
- Reference the topics: Education, Economy, Government, Social Life, Health or COVID-19 related statics (infection rates and death records).
- Be written in Modern Standard Arabic (MSA) or Moroccan dialect (MD).

It is worth pointing out that, comments written in French or English were excluded from our study given that their number is insufficient to construct a representative sample.

**Figure 2:**
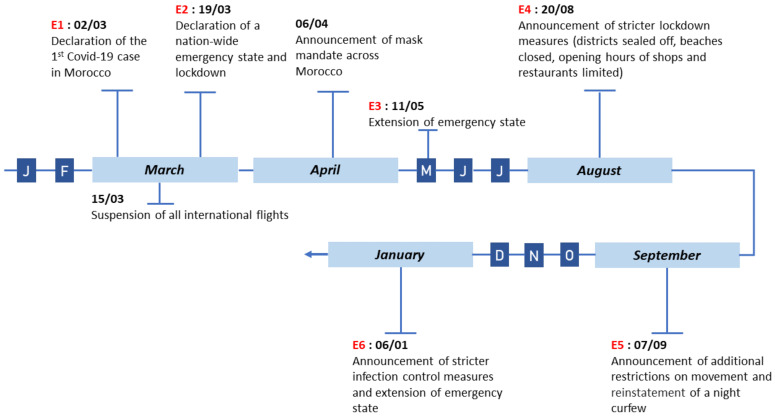
An excerpt of the timeline of COVID-19 related events in Morocco.

In addition to social media and digital press comments, we constructed a timeline of COVID-19 events including government decisions regarding the pandemic as well as the evolution of sanitary measures put in place for mitigation purposes. This event collection was based on a number of official government communications and press releases and were gathered from various sources such as: the official website of the Prime Minister of Morocco, the official website of the General Secretary of Government, the official website of the General Confederation of Moroccan Businesses (CGEM) and the official website of the Ministry of Economy and the Economic Watch Committee. An excerpt of the data collected on these events and their evolution is described in Figure 2.

#### 3.1.2. Data Annotation

The analysis of various random samples of our dataset yielded many recurrent topics. Upon studying the socio-economic effects of the pandemic described in literature reviews [26], we formalized a set of 7 classes representing topics of interest:

- **Education**: relating to any educational aspect from e-learning in schools and universities to postponing midterms and national examinations.
- **Economy**: relating to unemployment rates, poverty rate, economic crisis and quality of life.
- **Statistics**: relating to the number of cases and mortality rate reported on COVID-19 in Morocco.
- **Government**: relating to the political sphere, head of government, legislation, house of representatives and house of councilors.
- **Health**: relating to medication prescribed to COVID-19 patients, usage of Hydroxychloriquine and COVID-19 vaccines.
- **Sanitary measures**: relating to lockdown, quarantine, mask mandate, emergency state and contact tracing in Morocco.
- **Social Life**: relating to the impact of COVID-19 on different aspects of social life such as mosques, restaurants, public gatherings and group activities.

These topics of interest were then used as labels for supervised topic classification.

The Plutchik theory [27] is a psychoevolutionary theory of emotion (PTE) which classifies general emotional responses into eight categories: joy, sadness, anger, fear, trust, disgust, surprise and anticipation. The literature on Artificial Intelligence driven emotion classification has been making use of these categories as the basis for multi-label emotion detection [28].

Many studies have adapted these categories to specific contexts depending on the nature of the event studied, often supplementing them with additional categories such as optimism, pessimism and love. In the case of COVID-19, studies either focus on the resulting emotional impact of the pandemic from a psychological standpoint [29], or, as in the case of [30], the sentiments evoked by headlines related to the pandemic. The findings of [30] summarize the emotions linked to COVID-19 headlines in 8 categories: anger, anticipation, disgust, fear, joy, sadness, surprise and trust.

Upon analyzing random samples of our dataset, studying the literature on emotion analysis and taking into consideration the characteristics of the pandemic, a consensus has been reached between team members on the use of the 6 following emotions:

- **Optimism**: expression of hopefulness and positivity towards the outcome of the pandemic. *This emotion can be conveyed through faith based hope, science based belief or in the form of trust in government actions.*
- **Approval**: expression of agreement with government legislation and containment and mitigation measures deployed to curb the spread of COVID-19.
- **Mistrust**: expression of suspicions, wariness and doubts towards COVID-19, reported news or government decisions.
- **Anger**: expression of annoyance and hostility towards the situation.
- **Sadness**: expression of sorrow towards the situation.
- **Fear**: expression of panic, fright and dread towards the situation.

These emotions were then used as labels for supervised emotion classification.

Positive emotions (Optimism and Approval) and Negative emotions (Mistrust, Anger, Sadness, Fear) were aggregated into two classes for polar sentiment classification.

### 3.2. Moroccan Dialect Universal Language Model (MD-ULM)

The Moroccan Dialect (MD) is a low-resource language. Consequently, producing accurate text classification tasks such as sentiment analysis and emotion recognition is challenging. However, advances in Transfer Learning have recently shown promising results for improving text classification accuracy for both English [31] and Arabic [32]. These models are generally pre-trained on large unlabeled corpora and then fine-tuned on task-specific datasets. Universal Language Models, in particular, have achieved state-of-the-art results in various NLP tasks in English. The work described in [32] further illustrates that similar success can be achieved for Arabic.

Despite the lack of resources in Moroccan Dialect, there is a considerable amount of untapped potential in unlabeled data from social media comments and posts, websites for novels and short stories and Moroccan Wikipedia articles. These data sources can be exploited in order to yield more accurate NLP models.

In what follows, we present the first Universal Language Model for the Moroccan Dialect ‘MD-ULM’ (Moroccan Dialect Universal Language Model). Additionally, we illustrate that MD-ULM outperforms classical machine learning classification methods for the Moroccan dialect on three different tasks: Topic classification, Emotion Recognition and Polar Sentiment Analysis.

Using MD-ULM presents a considerable advantage: once the model is pre-trained, the learning process becomes limited to learning the parameters of the additional task-specific layers through transfer learning. The complete MD-ULM architecture consists of the combination of a pre-trained ULM model and additional task-specific layers for the desired tasks.

In order to develop our language model, we follow the general pipeline used in [32] with one major distinction in the pre-training corpora. In [32], the authors use Wikipedia articles. In our work, due to the scarcity of Wikipedia articles written in Moroccan Dialect, we target multiple and diverse sources to collect data. This step is technically challenging and time consuming since it requires several pre-processing steps.

**Figure 3:**
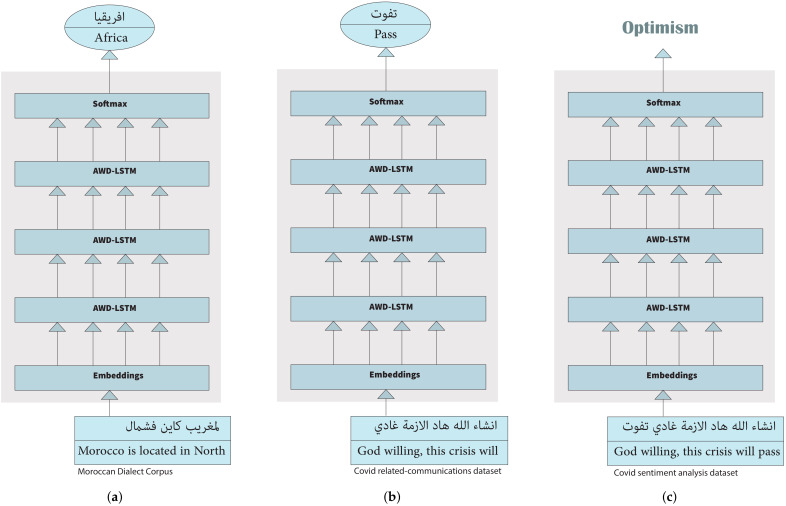
Three-step process for creating MD-ULM. (**a**) General Domain Language Model Pre-training (MD-ULM Pre-training). (**b**) Target Task Language Model Fine-tuning (MD-ULM fine-tuning). (**c**) Target Task Classification.

As illustrated in Figure 3, MD-ULM consists of three main stages: (i) pre-training the widely used language model AWD-LSTM [33] architecture, (ii) fine-tuning the pre-trained language model on a target dataset and (iii) adding a classification layer on top of the fine-tuned language model for text classification.

(i) General Purpose Pre-Training of MD-ULM

In order to capture the various properties of the complex morphology of the Moroccan Dialect, we constructed a large-scale language Modeling dataset. This dataset was built by scraping MD text data from Facebook, Twitter, Moroccan news websites, Moroccan Youtube channels, websites for books and stories written in Moroccan Dialect, Moroccan forums and Wikipedia articles.

The pre-processing step mainly consisted of extracting raw text data resulting from the scraping process and removing components written in non-Arabic letters. It is worth noting that we opted to not remove non-Arabic words if they appear in the context of a sentence to avoid altering the overall meaning. The constructed dataset was used to train three layers of the AWD-LSTM architecture. The output of this step is the model weights and word embeddings of each word in the constructed corpus.

(ii) MD-ULM Fine-Tuning on COVID-19 Related Communications

Even though the general-purpose dataset is diverse, COVID-19 related communications are more likely to follow a slightly different distribution. Therefore, this step is dedicated to fine-tuning MD-ULM on COVID-19 related communications.

To that end, we construct a language modeling dataset for COVID-19 following the same approach discussed in (i) with the exception of using specific COVID-19 related keywords in the scraping requests. In this step, we use discriminative fine-tuning (i.e., using different learning rates for different layers), which is known for its efficiency [34] since different layers capture different types of information.

(iii) Adding Classification Task-Specific Layers to MD-ULM

This step consists of adding two fully-connected layers to the language model for classification with ReLu and Softmax activations, respectively. In order to do so, we first freeze all previous layers and then train the two fully connected layers from scratch. Next, the lower frozen layer is unfrozen and fine-tuned until convergence after each epoch in a gradual fine-tuning manner, allowing us to avoid any loss of the information captured during language modeling. Finally, we evaluate the performance of MD-ULM using three conventional Machine Learning methods as a baseline, namely, Multinomial Logistic Regression Classifier, Naïve Bayes’ Classifier and Support Vector Machines. Table 1 illustrates the performance of our Moroccan Dialect Universal Language Model for the tasks of topic modeling, emotion classification and polar sentiment classification.

## 4. Results and Discussion

In this section, we present our findings on the evolution of COVID-19 social media comments, in terms of expressed emotions and discussed topics in time and through various COVID-19 related events. Then, we will discuss the limitations of our work and future improvements.

### 4.1. Evolution of COVID-19 Social Media Comments and Emotions

**Figure 4:**
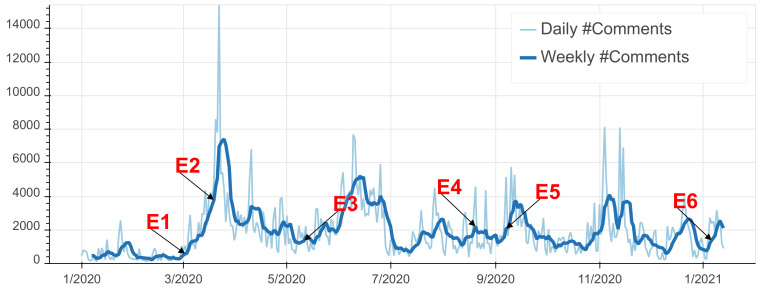
Temporal evolution of COVID-19 related social media communications in Morocco.

As illustrated in Figure 4, the total number of COVID-19 related social media comments hit an all-time peak at the end of March 2020. This concurs with the declaration of a public health emergency state (cf. Event E2 in Figure 2) accompanied with governmental sanitary measures such as a nation-wide lockdown and an inter-city ban of traffic, all due to the apparition of COVID-19 cases in Morocco exceeding 100 positive cases. In addition, we observe a spike in the number of comments around the end of June 2020. This is due to the official reports of Morocco’s fast increase of COVID-19 cases reaching 10,000 positive cases in 24 June.

**Figure 5:**
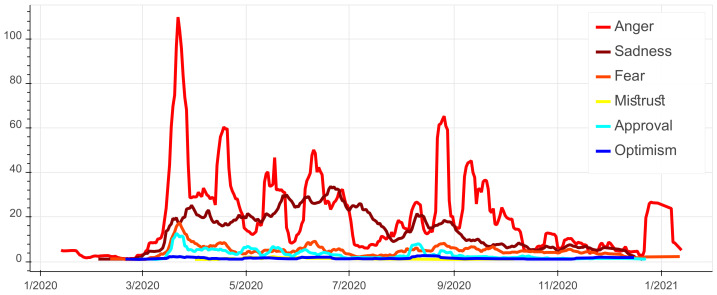
Summary of evolution of emotions in time.

**Figure 6:**
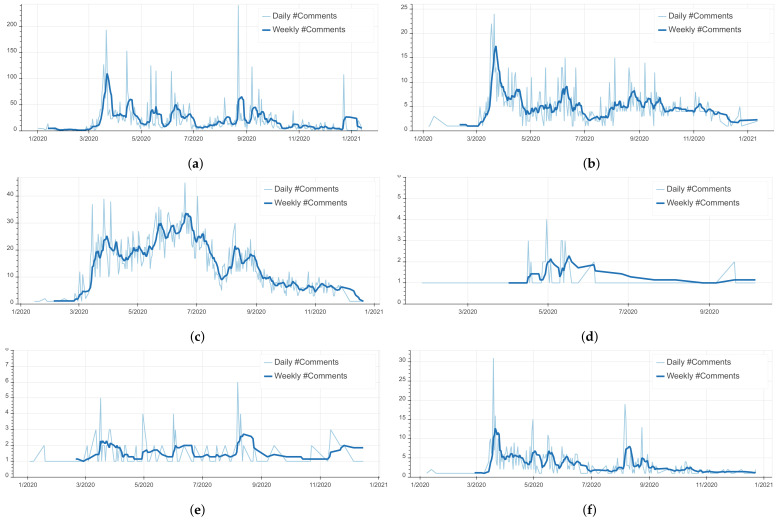
Evolution of emotions in time (Number of comments/Time). (**a**) Evolution of ‘Anger’ in time. (**b**) Evolution of ‘Fear’ in time. (**c**) Evolution of ‘Sadness’ in time. (**d**) Evolution of ‘Mistrust’ in time. (**e**) Evolution of ‘Optimism’ in time. (**f**) Evolution of ‘Approval’ in time.

As for the Moroccan’s expression of emotions, we observe in Figure 5 that all the emotions are correlated with the number of comments with a clear dominance of ‘Anger’ and ‘Sadness’ compared to the other emotions. This is perfectly in line with the results of similar work carried out in other countries such as Brazil and Spain [18,35]. For the detailed evolution of each emotion in time please refer to Figure 6. The peak of ‘Anger’ can be observed during the beginning of the outbreak. By investigating the comments, we found that the preemptive sanitary measures were met with discontent as they were deemed unnecessary by the public because of the low infection rate at the time. Furthermore, we noticed that Moroccans constantly express their ‘Anger’ with the announcements of infection rates’ increases and governmental sanitary countermeasures in the news platforms. This is manifested in Figure 5 by the series of peaks such as the ones around mid-April caused by the announcement of mask mandate and around the end of June and the beginning of August due to the announcement of stricter lockdown measures (cf. Figure 2 E4).

As for ‘Sadness’, we observe a stagnation between March 2020 and June 2020. By investigating the comments, this emotion is closely related to the rise of fatalities. Around December 2020, and after 10 months of COVID-19, online expressions of sadness began to drop. This is in fact associated with the emergence of new information about vaccines as well as emotional fatigue and the public’s desensitization to the number of cases and deaths.

Moreover, expressions of ‘Fear’ remained consistently strong throughout 2020 but have been especially high during the reporting of record numbers of positive cases and fatalities. The comments show that ‘Fear’ has also surrounded the devastating impact of COVID-19 on the healthcare system, the economy and the educational system.

Concerning ‘Mistrust’ and ‘Optimism’, they have been expressed differently by Moroccan social media users. While ‘Optimism’ remained, on average, almost consistent throughout 2020, ‘Mistrust’ marked mostly the months of April, May and June. Around that time, the Moroccan authorities were arresting fake news publishers and spreaders. ‘Mistrust’ sometimes surrounds the veracity of the existence of COVID-19, the government’s decisions and the safety of the vaccines. The latter can be seen in the spike after September 2020. ‘Optimism’ remained present in online chatter about COVID-19 in Morocco, reflecting a consistent hopefulness for a post-pandemic life. The comments show that the sentiment of ‘Optimism’ is manifested by religious beliefs and faith in science and by trust in governmental decisions.

Finally, ‘Approval’ illustrates trust in the government and is reflected during the important dates in which the government announced various sanitary measures (e.g., masks, lockdown, curfew and social distancing), especially the preventive measures taken in the beginning of the pandemic.

### 4.2. Distribution of Comments by Topic

In the following, we delve into the analysis of COVID-19 topics discussed by Moroccan citizens in terms of frequency and dominant keywords.

**Figure 7:**
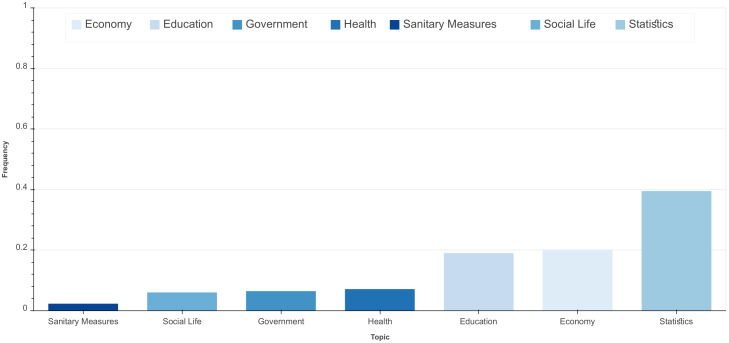
Distribution of topics over documents.

**Figure 8:**
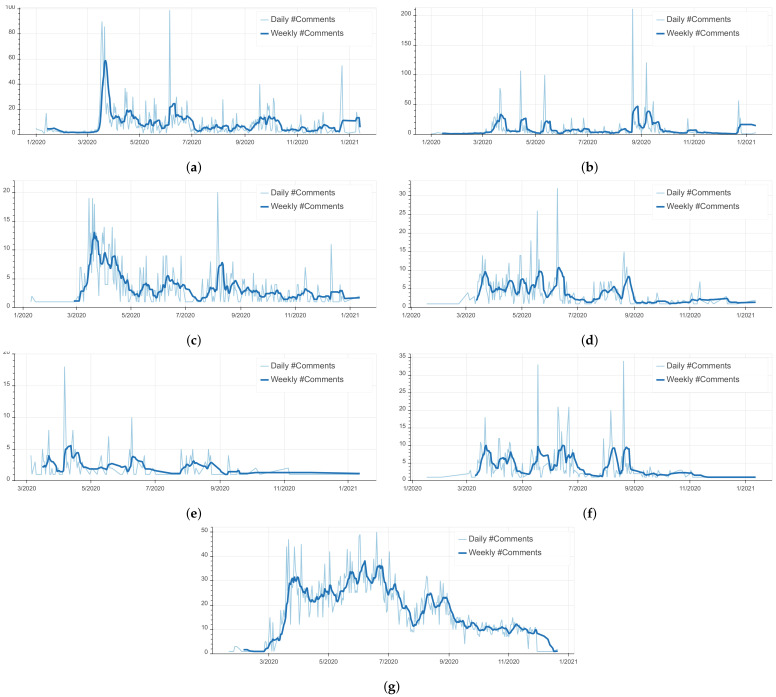
Evolution of topics in time (Number of comments/Time). (**a**) Evolution of ‘Economy’ in time. (**b**) Evolution of ‘Education’ in time. (**c**) Evolution of ‘Health’ in time. (**d**) Evolution of ‘Government’ in time. (**e**) Evolution of ‘Sanitary Measures’ in time. (**f**) Evolution of ‘Social Life’ in time. (**g**) Evolution of ‘Statistics’ in time.

Figure 7 illustrates the frequency of COVID-19 related comments by discussed topics. As shown, the most dominant topic is ‘Statistics’. Given the public’s attention to the number of cases and fatalities, the dominance of this topic is expected. The topics ‘Economy’ and ‘Education’ are the second and third most frequently discussed COVID-19 related topics online. These two aspects have been deeply affected by the current pandemic. Reviewing comments related to the topic ‘Economy’ showed that Moroccans are concerned with the impact of sanitary measures specifically on small businesses and informal sectors. In addition, the predominant and recurrent discussions related to ‘Education’ show their manifestation of confusion about the educational measures taken by the government and their demands for adapting the educational system to the requirements of remote e-learning, a measure that has been substantially challenging but ultimately a beneficial step in the mitigation and control of COVID-19. Figure 8 provides a detailed overview of the temporal evolution of topics discussed on social media. It is worth noting that the spikes in the volume of discussions about a specific topic reflects in general Moroccans’ follow-up of published news and government announcements on that topic.

**Figure 9:**
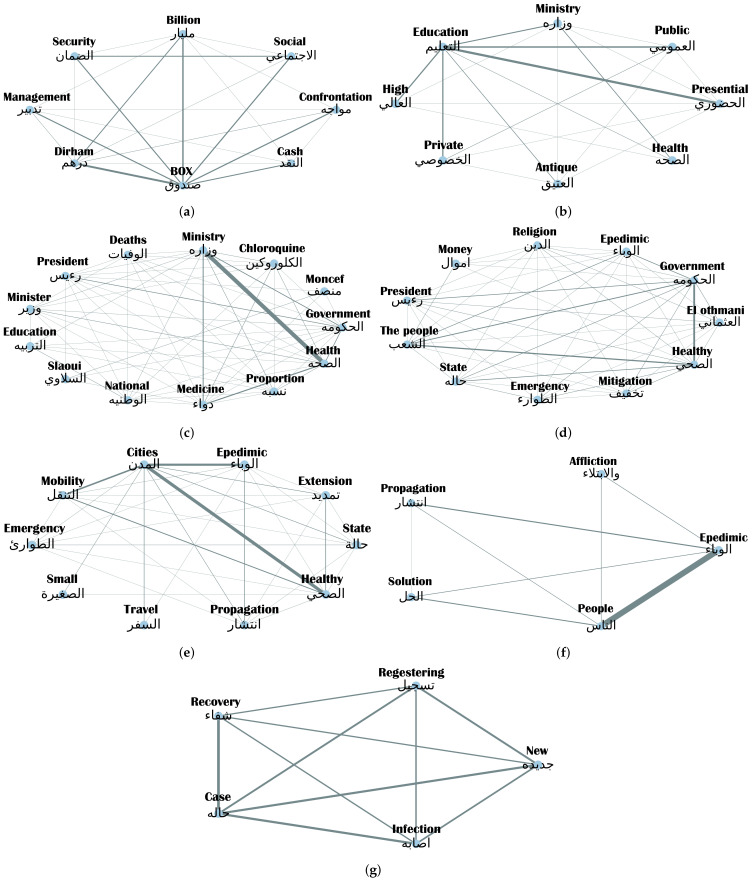
Connections between the most dominant words in COVID-related communications in Moroccan social media. Thicker lines correspond to higher co-occurrences. (**a**) Connections between the most dominant words for ‘Economy’ related communications. (**b**) Connections between the most dominant words for ‘Education’ related communications. (**c**) Connections between the most dominant words for ‘Health’ related communications. (**d**) Connections between the most dominant words for ‘Government’ related communications. (**e**) Connections between the most dominant words for ‘Sanitary Measures’ related communications. (**f**) Connections between the most dominant words for ‘Social Life’ related communications. (**g**) Connections between the most dominant words for ‘Statistics’ related communications.

**Figure 10:**
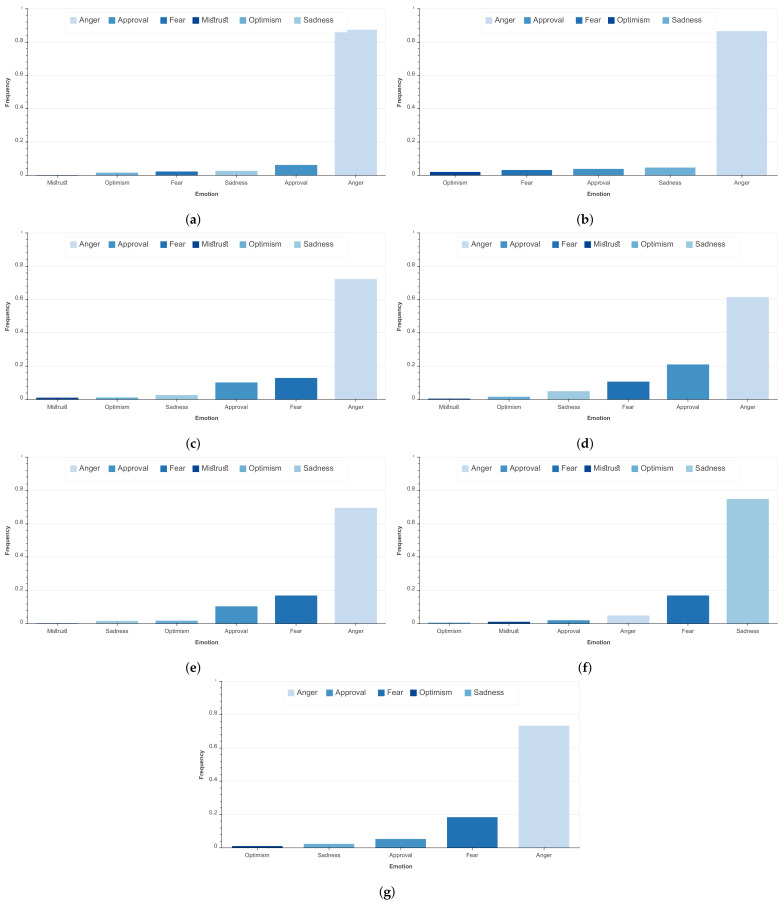
Distribution of emotions over the identified topics. (**a**) Distribution of emotions over the topic ‘Economy’. (**b**) Distribution of emotions over the topic ‘Education’. (**c**) Distribution of emotions over the topic ‘Health’. (**d**) Distribution of emotions over the topic ‘Government’. (**e**) Distribution of emotions over the topic ‘Social Life’. (**f**) Distribution of emotions over the topic ‘Statistics’. (**g**) Distribution of emotions over the topic ‘Sanitary Measures’.

Figure 9 illustrates the co-occurrence frequency of each topic’s most dominant words. In the topic of ‘Economy’ (Figure 10a), the thickest connecting line highlights the co-occurrence of the word ‘Box’ and the word ‘Dirham’ (the official monetary currency of Morocco). The pairing of these two words is in fact referencing the Special Fund for Coronavirus Management and Relief, established on March 15th to upgrade health infrastructure and support the worst-affected sectors of the economy. By the end of the same month, it had raised 18.3 billion Dirhams (≈2 million USD), making it a popular topic of discussion online.

To ensure continuity in teaching and learning during lockdown, schools and universities provided interactive online courses and e-learning platforms. In late August 2020, The Ministry of Education enabled students and their parents to choose their preferred mode of education for the upcoming academic year and 80% of them opted for in-person classes. This preference is reflected in the high co-occurrence of the words “Education” and “Presential” in the topic of “Education” (Figure 10b).

In the topics of ‘Health’ and ‘Government’ (Figure 9c,d), online comments mainly referenced the Ministry of Health and Head of Government (Saad-eddine El Othmani), respectively. Figure 10c,d highlight the ‘Anger’ expressed by social media users as a response to these topics. Some ‘Approval’ is expressed towards the topic of Government, which may be linked to the alleviation of restrictions or to the public’s agreement with the crisis mitigation strategy.

The most dominant words in the topic of ‘Sanitary Measures’ include: mobility, travel, cities and propagation (Figure 9e), highlighting the interest of social media users in COVID-19 regulations. This, in addition to ‘Anger’ being the most dominant reaction to this topic as well as the topic of “Social Life” (Figure 10e), allows us to stipulate that social media users are reacting negatively to the restrictions of travel between cities and the lack of ability to move freely without risk of infection and viral propagation. As can be expected, ‘Sadness’ is the most expressed emotional reaction to the topic of “Statistics”, with ‘recovery’, ‘case’, and ‘infection’ being the most widely used words in comments related to this topic (Figure 9g).

### 4.3. Distribution of Emotions by Topics

Figure 10 illustrates the distribution of emotions by identified topics. As shown, the most predominant emotion over all topics is ‘Anger’, except for the topic ‘Statistics’ where most comments express the ‘Sadness’ emotion followed by the ‘Fear’ emotion. The latter is in fact related to people expressing their sadness and fear, usually related to the increase in the number of positive cases and fatalities. The ‘Anger’ emotion dominance is more pronounced for topics ‘Economy’ and ‘Education’. By investigating the comments, Moroccans were concerned with the impact of the strict sanitary measures on the economy, especially the economic recession that touched the medium and small enterprises as stated earlier. Moreover, noticeable is the ‘Approval’ emotion ranked second in the ‘Government’ topic. This represents a relatively important proportion of comments when compared to emotions other than ‘Anger’. Finally, ‘Optimism’ and ‘Mistrust’ remain the least represented emotions over all topics.

### 4.4. Limitations

Our work has implications for the research community in Morocco, as well as practical implications for health operators in their efforts for an improved and active monitoring of discussed topics and induced emotions related to the special case of COVID-19 and to pandemics in general. However, our study suffers from some limitations such as the access to high quality data that stems essentially from limited access to data sources. In addition, it is worth mentioning that a portion of the Moroccan population does not have access to or use social media platforms due to digital illiteracy. This lack of data excludes the portion of Moroccans highly impacted by the COVID-19 pandemic, especially at the economic and educational levels.

## 5. e-Covid-19: A Moroccan Platform for COVID-19 Management

**Figure 11:**
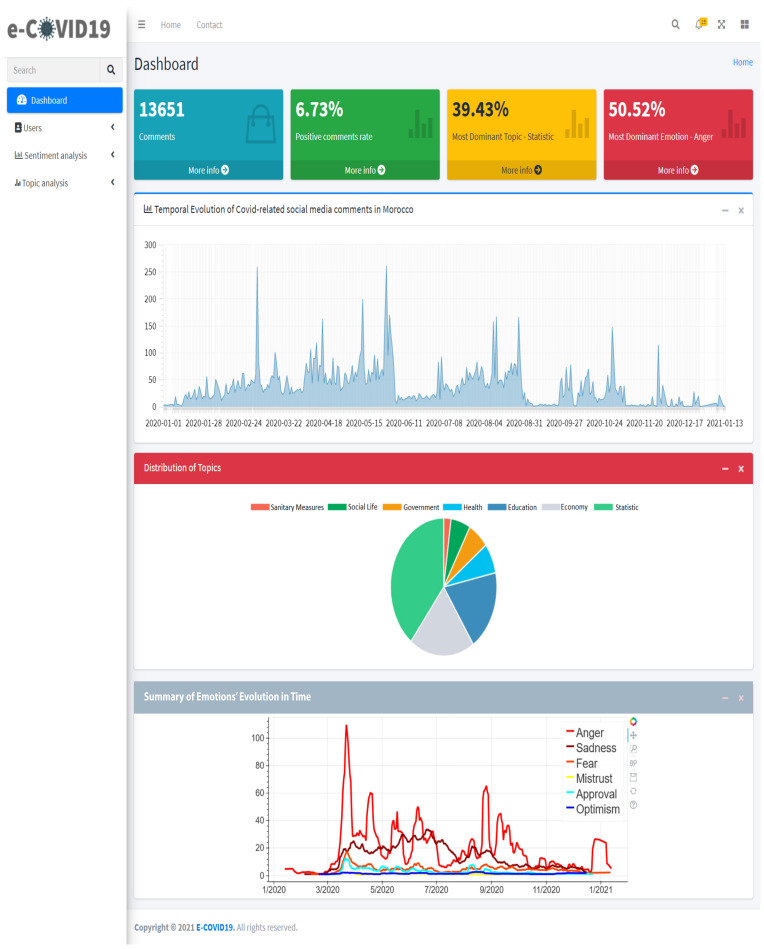
Moroccan platform for a better management of COVID-19 pandemic.

In this section, we present our proposed decision-making assistance tool and platform: e-Covid19. This Artificial Intelligence based platform aims to provide real-time social media analysis, automated sentiment analysis and topic modeling. A view of the platform’s dashboard is illustrated in Figure 11.

The e-Covid19 platform offers several functionalities freely available for three types of users: *Administrator*, *User* and *Visitor*.

Users (government entities, Moroccan interior ministry, etc.) and administrators can access and view:

- Temporal evolution of COVID-19 related social media comments.
- Detailed statistics and graphs on emotion analysis during COVID-19: Number of comments by emotion, evolution of emotions in time and distribution of emotions over topics.
- Detailed statistics and graphs about topic analysis during COVID-19: Number of comments by topic, distribution of topics and evolution of topics in time.
- Filtered results by date.
- Detailed description of the source, topic class and emotion class of comments, with the possibility to extract this data in various formats (e.g., .csv, .pdf, .xlsx).

Administrators can also create user accounts and view usage statistics such as the number of users and visitors. Visitors, which represent any citizen, can view public statistics such as percentages of comments by topic and by emotion, as well as the most dominant topics and the collective emotional response. All the data provided by the e-Covid19 platform will be anonymous; no information about the identity of the people who emit the messages will be available.

The e-Covid19 platform allows for regular daily data collection from the social media websites and news outlets included in our study. It provides real-time sentiment and emotion analysis as well as topic modeling for the Moroccan Dialect. This platform is put in service for COVID-19 mitigation and allows decision makers to adjust regulations in real time based on the observed responses and to launch targeted awareness campaigns based on which issues are met with the most mistrust or anger and which topics capture the interest of social media users. The use of platform will be free to visitors.

Despite social media users not representing the entirety of Moroccan citizens, their role in spreading awareness is of utmost importance. Capturing their emotional response to various government and public health regulations allows decision makers to devise a social media strategy aiming to educate and communicate with social media users and would eventually be influential in understanding how the public reacts to COVID-19 and finding ways to contain situations of tension and unresponsiveness to mask mandates, vaccination campaigns and possible recurrent lockdowns.

## 6. Conclusions

The COVID-19 pandemic has incited millions of social media users worldwide to share their thoughts, fears and criticism. A widespread outbreak of this viral disease has had a considerable impact on Morocco, its economy, its tourism sector, its educational system and its citizens’ social life. To study the emotional response of Moroccan social media users to the many aspects COVID-19 continues to affect, we developed a methodology aiming to acquire comments from different data sources such as social media websites (Twitter, Facebook, Youtube) and news outlets (Hespress, Hibapress) and label a subset of them based on which topic each comment discusses and which emotion its writer expresses. Using this annotated data, we use our newly proposed Moroccan Dialect Universal Language Model, fine-tune it for text classification and score its accuracy against baseline supervised learning methods. As a result, we are able to classify comments by emotion and topic as well as to display their evolution in time.

The results inferred from this study provide major insights into how Moroccan social media users reacted to various marking events related to COVID-19 in 2020 and illustrate the impact of effective government-citizen communication in mitigating the pandemic, surviving 2020 and attempting to thrive in a post-pandemic economic crisis.

As a future work, from a technical perspective, it would be valuable to reinforce our language model with the inclusion of other North-African dialects. For instance, Algerian and Tunisian dialects grammatically resemble the Moroccan dialect and can enlarge the training corpus and vocabulary spectre of the MD-ULM input space. It would also be useful to assess the extent to which multitask learning using semantically similar dialects could improve the accuracy of the MD-ULM in inferring emotions and topics.

Moreover, the work described in this paper can potentially be combined with community detection methods in order to determine if belonging to a specific group affects a user’s emotional response. Sentiment analysis by topic provides a precise snapshot of the sentiment distribution in a social network, potentially allowing the identification of communities or sub-units of users within the network.

## Figures and Tables

**Table 1:** Performance results for Emotion, Topic and Polarity classification.

	Emotion	Topic	Polarity
Model	Accuracy		
MultinomialNB	0.31	0.51	0.64
Logistic Regression	0.33	0.53	0.61
SVM	0.33	0.59	0.65
MD-ULM	**0.43**	**0.70**	**0.70**
